# Pentraxin-3 and C-reactive protein plasma levels predict survival in older adults with or without metabolic syndrome – results of the PolSenior2 substudy

**DOI:** 10.1186/s12979-025-00509-9

**Published:** 2025-05-08

**Authors:** Aleksander J. Owczarek, Anna Ochman, Anna Chudek, Małgorzata Mossakowska, Monika Puzianowska-Kuźnicka, Hanna Kujawska-Danecka, Tomasz Zdrojewski, Andrzej Więcek, Jerzy Chudek, Magdalena Olszanecka-Glinianowicz

**Affiliations:** 1 Health Promotion and Obesity Management Unit, Department of Pathophysiology, Faculty of Medical Sciences in Katowice, Medical University of Silesia in Katowice, Katowice, Poland; 2 Department of Internal Medicine and Oncological Chemotherapy, Faculty of Medical Sciences in Katowice, Medical University of Silesia in Katowice, Katowice, Poland; 3 Study on Aging and Longevity, International Institute of Molecular and Cell Biology, Warsaw, Poland; 4 Department of Human Epigenetics, Mossakowski Medical Research Institute, Polish Academy of Sciences, Warsaw, Poland; 5 Department of Geriatrics and Gerontology, Medical Centre of Postgraduate Education, Warsaw, Poland; 6 Division of Preventive Medicine and Education, Medical University of Gdansk, Gdansk, Poland; 7 Department of Nephrology, Transplantation and Internal Medicine, Faculty of Medical Sciences in Katowice, Medical University of Silesia in Katowice, Katowice, Poland

**Keywords:** Pentraxin 3, C-reactive protein, Aging, Survival, Population-based study

## Abstract

**Objective:**

There are no published data on the associations between plasma concentration of pentraxin-3 (PTX-3) - a marker of vascular inflammation and mortality in older subjects with or without metabolic syndrome (MS). Therefore, we aimed to compare the prognostic significance of increased PTX-3 and CRP levels on overall survival in subjects aged 60 and older with and without MS.

**Materials and methods:**

Study participants (*N* = 3534) were categorized according to the presence or absence of MS and then each of these groups was stratified into 3 subgroups based on concentrations of CRP (≤ 3 mg/dL and > 3 mg/dL) and PTX-3 (< and ≥ the sex-specific cut-off values, based on the ROC curve analysis with the Youden index): double-negative inflammatory markers (low CRP and PTX-3 plasma concentrations); single-positive inflammatory marker (increased CRP or PTX-3 plasma concentrations) and double-positive inflammatory markers subgroup (increased CRP and PTX-3 plasma concentrations). During the 4.19-year follow-up, 678 (19.2% of the entire cohort) individuals died including 401 men (22.9%) and 277 women (15.5% ).

**Results:**

The optimal cut-off for PTX-3 plasma concentration associated with an increased risk of death was 2.07 ng/mL for men and 2.23 ng/mL for women. The death rates were increased for single-positive and were highest in double-positive subgroups both for men and women, with or without MS. Kaplan-Meier analysis showed no effect of MS on survival in men and women in subgroups within specific inflammatory marker categories. Of note, the inflammatory markers class effect on survival was already significant in the single-positive subgroups (34% and 44% higher risk for death for men and women), and even more pronounced for the double-positive subgroup (more than two and almost three times higher risk of death for men and women, respectively). In the entire study group, a weak correlation was found between plasma concentrations of PTX-3 and hs-CRP (ρ = 0.11, *p* < 0.001) and slightly higher in undernourished subjects with hs-CRP > 3 mg/dL (ρ = 0.28, *p* < 0.001).

**Conclusion:**

Our study suggests that in the age-advanced Caucasian population, the inflammatory status with increased plasma levels of both PTX-3 and CRP is associated with a higher risk of all-cause mortality, regardless of the occurrence of MS. However, due to the retrospective study design, these results require confirmation in prospective studies with an analysis of the underlying causes of death.

**Supplementary Information:**

The online version contains supplementary material available at 10.1186/s12979-025-00509-9.

## Introduction

A meta-analysis of 87 studies including 951,083 patients with metabolic syndrome (MS) showed that MS is associated with a 2-fold increase in cardiovascular (CV) outcomes and a 1.5-fold increase in all-cause mortality [1]. Similarly, a meta-analysis of 20 studies, including 54,661 subjects aged 65 years and older, found that MS is associated with moderately increased CV and all-cause mortality. Also, in this analysis, elevated blood glucose and decreased HDL cholesterol levels were associated with significantly increased mortality [2]. Of note, visceral obesity, which is one of the components of MS, is the cause of systemic microinflammation [3], and atherosclerosis is considered an inflammatory disease associated with all components of MS [4].

Pentraxin-3 (PTX-3) has been suggested to be a more specific marker of vascular inflammation than other members of the pentraxin family, including C-reactive protein (CRP) [5]. CRP, a short pentraxin, is produced mainly by the liver in response to increased interleukin-6 levels. In contrast, the long one, PTX-3, is produced by the vasculature and heart in response to primary inflammatory stimuli [6, 7]. It has also been shown that macrophages, endothelial, and smooth muscle cells in human atherosclerotic lesions produce PTX-3 [8]. In turn, PTX-3 stimulated synthesis of proinflammatory cytokines by macrophages that may further exacerbate endothelial dysfunction and affect the stability of the atherosclerotic plaque. PTX-3 inhibits endothelial nitric oxide synthase and activates inducible nitric oxide synthase that may enhance apoptosis. Furthermore, PTX-3 decreases the activity of antioxidant enzymes, elevates the GSH/GSSH ratio, and increases intracellular ROS concentration, thereby exacerbating intracellular oxidative stress [9]. Additionally, PTX3-induced neuropeptide Y synthesis promotes the uptake of lipids by vascular smooth muscle cells, leading to their transformation into vascular smooth muscle foam cells. Together, these data suggest that PTX-3 participates in the development of atherosclerosis and is considered a biomarker of vascular disorders [10]. In line with this hypothesis, worse CV outcomes after acute coronary events were accompanied by higher plasma PTX-3 levels, regardless of CRP [5, 11]. Moreover, higher plasma PTX-3 levels in patients with stable coronary heart disease (CHD) were associated with increased risk of all-cause mortality, CV events, and incident heart failure (HF) [12]. Furthermore, in a population-based study including men and women from four ethnic groups without clinically overt cardiovascular disease (CVD), plasma PTX-3 levels were associated with CV risk factors, subclinical CVD, coronary artery calcium score, and clinical CHD events [13]. In addition, higher plasma PTX-3 but not CRP correlated with future CV events in patients with HF with preserved ejection fraction (HFpEF) [14]. Another study showed that elevated plasma PTX-3 level was an independent risk factor for cardiac events in patients with chronic HF [15]. Higher plasma PTX-3 concentrations in patients with end-stage HF were associated with worse outcomes in addition to the increased NT-proBNP levels, lower serum sodium concentration, mean arterial blood pressure, BMI, and ischemic etiology of HF [16]. Three meta-analyses consistently confirmed an association between plasma PTX-3 levels and worse outcomes in coronary artery disease (CAD) patients. The first one including 9 studies and 5,174 patients with CAD showed that higher plasma PTX-3 levels could be an independent predictor of all-cause mortality, cardiac death, and cardiac events [17]. The second, including 16 studies and 11,007 patients with CAD found that higher plasma PTX-3 levels were associated with poor prognosis independently of CAD subtype, follow-up duration, and adjustment for CRP levels [18]. The third, including 15 studies and 11,365 participants, revealed a significant dose-dependent association between plasma PTX-3 concentration and the risk of poor outcomes in patients with CAD [19]. It is also important to note that in older adults without an established diagnosis of CVD, plasma PTX-3 levels were associated with CV and all-cause mortality regardless of plasma CRP concentration and CV risk factors including age, BMI, fasting glucose, lipids and insulin levels, blood pressure, physical activity and subclinical CVD measures [20]. Furthermore, some studies have shown positive associations between plasma PTX-3 levels and MS components and their severity [21–24]. Based on previous data we hypothesized that older subjects with MS have higher concentrations of PTX-3 and CRP than those without MS and therefore higher mortality. Nevertheless, to our knowledge, no data have been published on the associations between plasma PTX-3 levels and mortality in older subjects, with respect to MS. Therefore, this substudy of a large, nationwide, population-based project, aimed to evaluate the prognostic significance of increased PTX-3 and CRP levels for overall survival in subjects aged 60 and older with and without MS.

## Materials and methods

### Sub-study design

This analysis was a part of the PolSenior2 study – a large, population-based, nationwide interdisciplinary project conducted between January 2018 and December 2019. A multistage, stratified, and clustered sampling design was used for subjects aged 60 and older to obtain a sample representative for old and very old Polish citizens (almost exclusively Caucasians). In total, seven age groups of similar size and gender distribution (60–65, 65–69, 70–74, 75–79, 80–84, 85–89, and ≥ 90 years) were recruited according to the protocol described in detail previously [25]. The study procedures comprised structured surveys which included present health status, treated clinical conditions (diabetes, arterial hypertension), hospitalization for heart failure, coronary artery disease, acute myocardial infarction, stroke, Mini Nutritional Assessment-Short Form (MNA-SF), Instrumental Activities of Daily Living Scale (IADL), current medication use and socioeconomic data, and anthropometric and blood pressure measurements performed at home during three visits (https://polsenior2.mug.edu.pl).

**Fig. 1:**
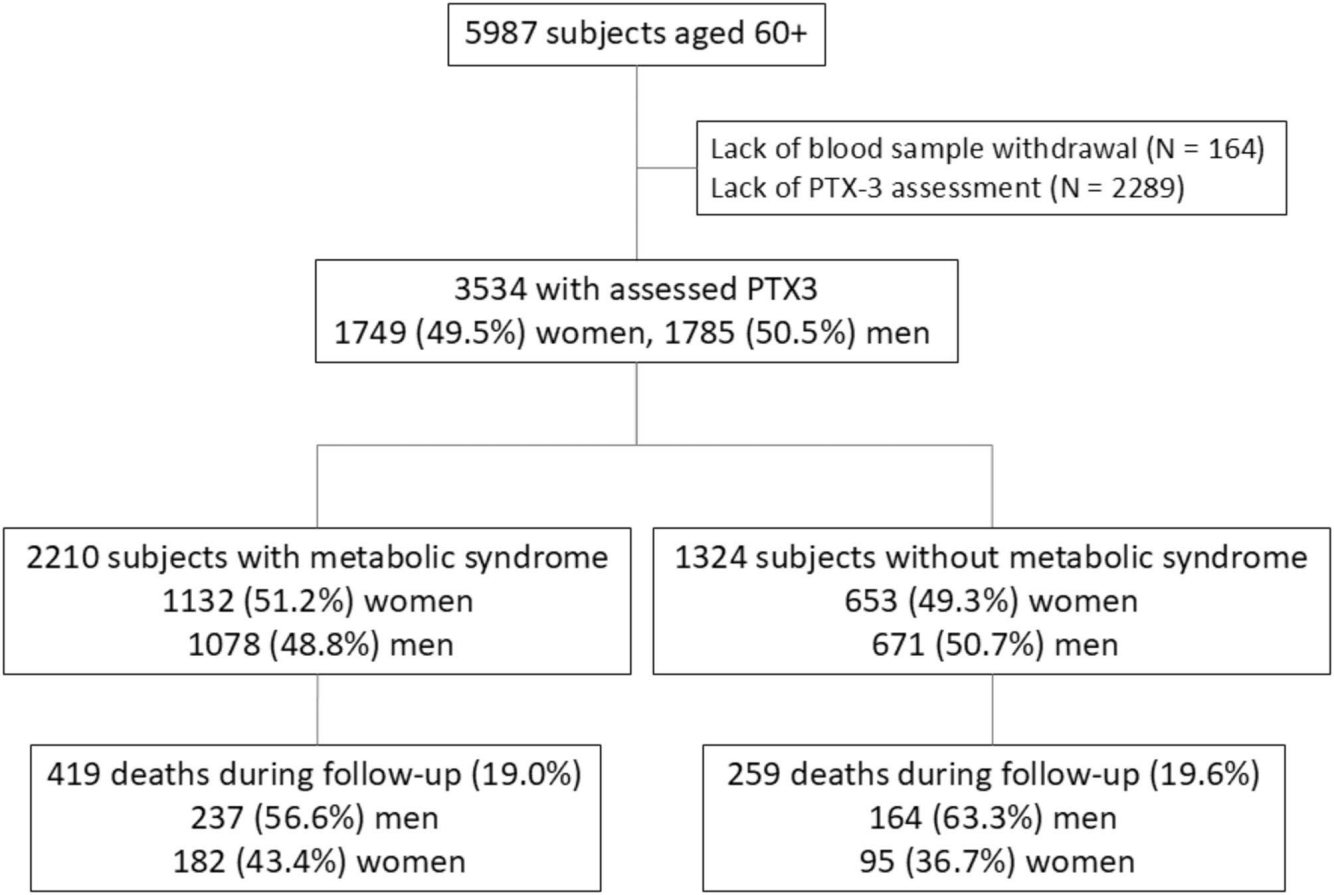
The study flow chart

In line with STROBE guidelines, the flow chart (Fig. 1) described that due to the lack of blood sample withdrawal (*N* = 164) or missing biobank plasma samples (*N* = 2289), 3534 out of 5987 individuals were included in this substudy based on PTX-3 assessments performed in the biobank plasma samples stored for 2 years from the date of collection at -70 °C.

All study subjects or their family caregiver gave written informed consent for participation, and all assessments were carried out according to the protocol approved by the Bioethics Committee of the Medical University of Gdansk (NKBBN/257/2017).

### Measurements

Laboratory tests included in the PolSenior2 protocol (serum total cholesterol, LDL cholesterol, HDL cholesterol, triglycerides, glucose, albumin, CRP) were assessed in a single, certified laboratory dedicated to the study (Bruss Laboratory in Gdynia) using automated systems (Siemens Healthcare) as previously described [25]. Plasma PTX-3 levels were quantified by the ELISA method (BioVendor, Brno, The Czech Republic) in the Department of Pathophysiology, Faculty of Medical Sciences, Medical University of Silesia in Katowice with the limit of quantification (LOQ) of 0.08 ng/mL and intra- and inter-assay coefficients of variations 6% and 12%, respectively.

### Survival analysis

Survival data were obtained from the Polish population register (PESEL). The total number of person-years of observation was 11,934.4. At the time of checking the register, 678 (19.2%) individuals included in this analysis had died including 401 (22.9%) men and 277 (15.5%) women with a death incidence rate of 56.8 (95% CI: 52.7–61.2) per 1000 person-years.

### Data analysis

Obesity was defined according to the WHO criteria based on BMI ≥ 30.0 kg/m^2^ [26]. Visceral obesity was assessed according to the International Diabetes Federation (IDF) using waist circumference cut-offs for Caucasians (≥ 94 cm for men and ≥ 80 cm for women) [27].

The diagnosis of MS was established according to the consensus definition presented in the 2009 joint interim statement of the International Diabetes Federation Task Force on Epidemiology and Prevention, the National Heart, Lung, and Blood Institute, the American Heart Association, the World Heart Federation, the International Atherosclerosis Society, and the International Association for the Study of Obesity [27]. Any three components present in an individual warranted the diagnosis of MS: (1) Visceral obesity for Europeans (IDF criteria), (2) Serum triglyceride level ≥ 150 mg/dL or treatment of hypertriglyceridemia, (3) Low serum HDL-cholesterol concentration (< 40 mg/dL in men and < 50 mg/dL in women) or treatment of this condition, (4) Systolic blood pressure ≥ 130 mm Hg or/and diastolic blood pressure ≥ 85 mm Hg or treated hypertension, (5) Fasting plasma glucose ≥ 100 mg/dL or using antidiabetic medication. Outside the criteria of MS, we classified subjects with prediabetes as those with fasting glucose levels between 100 and 125 mg/dL even using low doses of metformin (< 1 g daily) when the diagnosis of diabetes was not declared, in line with the guidelines of Polish Diabetes Association [28]. Other treated and those with fasting serum levels at least 126 mg/dl were considered to have diabetes.

The serum albumin level ≥ 40 g/L was considered a sign of good nutritional status [29]. In addition, we used MNA-SF as a screening tool for malnutrition [30]. Participants with 0–7 points out of 14 available points (indicating malnutrition) and 8–11 points (suggesting the risk of malnutrition) were pooled into one category (less than 12 points) as those with poor nutritional status (PNS).

CKD-EPI formula was used to estimate the glomerular filtration rate (eGFR) [31]. We applied age-adjusted criteria for eGFR, namely < 45 ml/min/1.73m^2^ as the cut-off for age-adjusted chronic kidney disease [32].

Functional status was analyzed using the IADL scale. Scores of 8–18 points were classified as a dependency, 19–23 points as a partial dependency, and 24 points as independence [33].

### Statistical analysis

The study participants were stratified according to the presence of MS (Fig. 1). All analyses were performed separately for men and women due to significantly different mortality rates. They were categorized according toplasma concentrations of CRP (≤ 3 mg/dL and > 3 mg/dL) and PTX-3 (less than sex-specific cut-off associated with increased mortality based on ROC analysis, and equal and more than the sex-specific cut-off) into 3 subgroups: with low CRP and PTX-3 (double-negative inflammatory markers subgroups), increased CRP or PTX-3 (single-positive inflammatory marker subgroups) and both markers increased (double-positive inflammatory markers subgroups).

Statistical analyses were performed using STATISTICA 13.0 PL (TIBCO Software Inc., Palo Alto, CA, USA), R software [R version 4.4.0 (2024-04-24 ucrt) – “Puppy Cup”, R Core Team (2021). R: A language and environment for statistical computing. R Foundation for Statistical Computing, Vienna, Austria]. Statistical significance was set at a *p*-value below 0.05. All tests were two-tailed. Imputations were not performed for missing data. Nominal and ordinal data were expressed as numbers and percentages. Interval data were expressed as median, with lower and upper quartiles. The distribution of variables was evaluated by the Anderson-Darling test and the quantile-quantile (Q–Q) plot. Nominal and ordinal data were compared with the χ2 test or Fisher test. Comparisons between groups for interval data were done with a non-parametric one-way analysis of variances (Kruskall test) with median post *hoc test* between groups. The rank Spearman correlation coefficient (also stratified) was used as a measure of associations between covariates. The sex-specific cut-off for PTX-3 associated with increased mortality was assessed for men and women based on the ROC curve analysis. Results were presented with the area under curve (AUC), sensitivity (Se), and specificity (Sp), all measurements with 95% confidence interval (CI). Overall mortality risk factors were assessed with the Cox proportional hazard models (univariate and multivariate). Results were presented with hazard ratios (HR) and 95% confidence intervals (CI) with corresponding *p*-values. The proportionality assumption was tested based on the Schoenfeld residuals (R function ‘*cox.zph*’). Multiple-collinearity was checked based on the correlation matrix of coefficients of the survival model. Additionally, overall survival analyses were done with Kaplan-Meyer curves, stratified by sex and inflammatory markers, with the pairwise comparison of survival curves and the Hochberg correction for multiple comparisons (R function ‘*pairwise_survdiff*’). Additionally, in each subgroup, the restricted mean survival times (RMST), representing the average survival time during a defined period ranging from time 0 to the end of the follow-up time, were calculated.

## Results

### Characteristics of the study population

Plasma PTX-3 concentrations were measured in 3534 subjects – 1749 women (49.5%). The study group was categorized according to the presence or absence of MS (Fig. 1).

We applied the ROC analysis to establish sex-specific cut-off points for plasma PTX-3 concentrations that are associated with an increased risk of death during the 4.19-year follow-up. The optimal cut-off was 2.07 ng/mL [AUC = 0.622, *p* < 0.001; Sensitivity (Se) = 65.7% and Specificity (Sp) = 55.0%] for men and 2.23 ng/mL (AUC = 0.645, *p* < 0.001; Se = 53.8% and Sp = 69.9%] for women – Table 1.

**Table 1:** Results of diagnostic study based on the PTX and CRP levels

	**Male**			
Estimation (± 95% CI)	PTX ≥ 2.07	CRP > 3.0	PTX + CRP Single-positive vs. double-negative	PTX + CRP Double-positive vs. double-negative
Sensitivity	65.7% (60.8–70.3%)	53.6% (48.6–58.6%)	67.2% (60.9–73.1%)	66.2% (59.8–72.2%)
Specificity	55.0% (52.3–57.7%)	68.0% (65.5–70.5%)	46.9% (44.0 – 49.9%)	70.8% (67.3–74.0%)
PPV	30.3% (27.2–33.4%)	33.3% (29.7–37.1%)	21.5% (18.6–24.6%)	41.7% (36.7–46.9%)
NPV	84.3% (81.7–86.7%)	83.1% (80.8–85.3%)	86.9% (83.9–89.5%)	86.9% (83.9–89.5%)
Accuracy	57.5% (55.1–59.8%)	64.7% (62.4–67.0%)	50.5% (47.9–53.2%)	69.7% (39.7–57.3%)
DOR	2.33 (1.85–2.94)	2.46 (1.96–3.09)	1.81 (1.35–2.43)	4.75 (3.48–6.49)
	**Female**			
Estimation (± 95% CI)	PTX ≥ 2.23	CRP > 3.0	PTX + CRP Single-positive vs. double-negative	PTX + CRP Double-positive vs. double-negative
Sensitivity	53.8% (47.7–59.8%)	50.2% (44.1–56.2%)	62.2% (54.9–69.0%)	53.5% (45.4–61.5%)
Specificity	69.9% (67.5–72.2%)	64.6% (62.1–67.0%)	51.7% (48.9–54.4%)	80.2% (77.3–82.8%)
PPV	24.7% (21.3–28.3%)	20.7% (17.7–23.9%)	15.7% (13.2–18.4%)	32.9% (27.2–39.1%)
NPV	89.2% (87.2–90.9%)	87.6% (85.5–89.5%)	90.4% (88.1–92.4%)	90.4% (88.1–92.4%)
Accuracy	67.4% (65.2–69.6%)	62.3% (60.0–64.6%)	53.0% (50.5–55.5%)	76.1% (73.3–78.6%)
DOR	2.70 (2.08–3.51)	1.84 (1.42–2.38)	1.76 (1.29–2.40)	4.45 (3.26–6.63)

PPV/NPV – positive/negative predictive value; DOR – diagnostic odds ratio

Study subjects were then stratified based on serum concentrations of CRP (≤ 3 mg/dL and > 3 mg/dL) and PTX-3 (< and ≥ the sex-specific cut-off values) into 3 subgroups: double-negative inflammatory markers subgroups (with low CRP and PTX-3 values); single-positive inflammatory marker subgroups (increased CRP or PTX-3 values) and double-positive inflammatory markers subgroups (increased concentrations of both markers) – Supplementary Tables 1–3. All analyses were performed separately for men and women categorized according to the presence of MS.

The study groups and subgroups for women and men were stratified into categories by MS and inflammation. Due to applied stratification, the median PTX-3 values were lowest in the double-negative subgroups, higher in the single-positive subgroups, and highest in the double-positive subgroups for both genders. The comparison of diagnostic study results between sex-specific cut-off for PTX-3 values, CRP < and ≥ 3.0 as well as single/double-positive vs. negative marker is presented in Table 1. For men, assessment based on PTX had higher sensitivity than CRP, yet lower specificity. The higher positive predictive value (PPV) and negative predictive value (NPV) were observed for double-positive cases. For women, assessment based on PTX had higher both sensitivity and specificity than CRP. Also here, the higher PPV and NPV were noted for a double-positive case.

Individuals from the single-positive subgroups were more likely to be older(aged > 80 yrs), dependent in IADL (< 24 pts), had poor nutritional status (MNA < 12 pts), lower albumin levels (< 40 g/L), and decreased eGFR (< 45 ml/min/1.73m^2^), but hypercholesterolemia was less common than subjects from the double-negative subgroup. Notably, the double-positive individuals were the oldest and less often independent, had the poorest nutritional status, and most frequently decreased eGFR and low albumin levels. At the same time, they the least often had hypercholesterolemia with the only exception of men with MS. Additionally, in subgroups with MS, the subjects from the single-positive subgroups more often had diabetes than individuals from the corresponding double-negative subgroups. Still, diabetes occurred most often in people from the double-positive subgroups. Of interest, the number of people with prediabetes was comparable in all subgroups stratified according to the inflammatory status.

### Outcome data

During a follow-up, the death rates per 1000 subjects for single-positive, and double-positive subgroups were increasing both for men and women, without and with MS, compared to double-negative. Of note, the highest rates were observed in double-positive subgroups without MS.

**Fig. 2:**
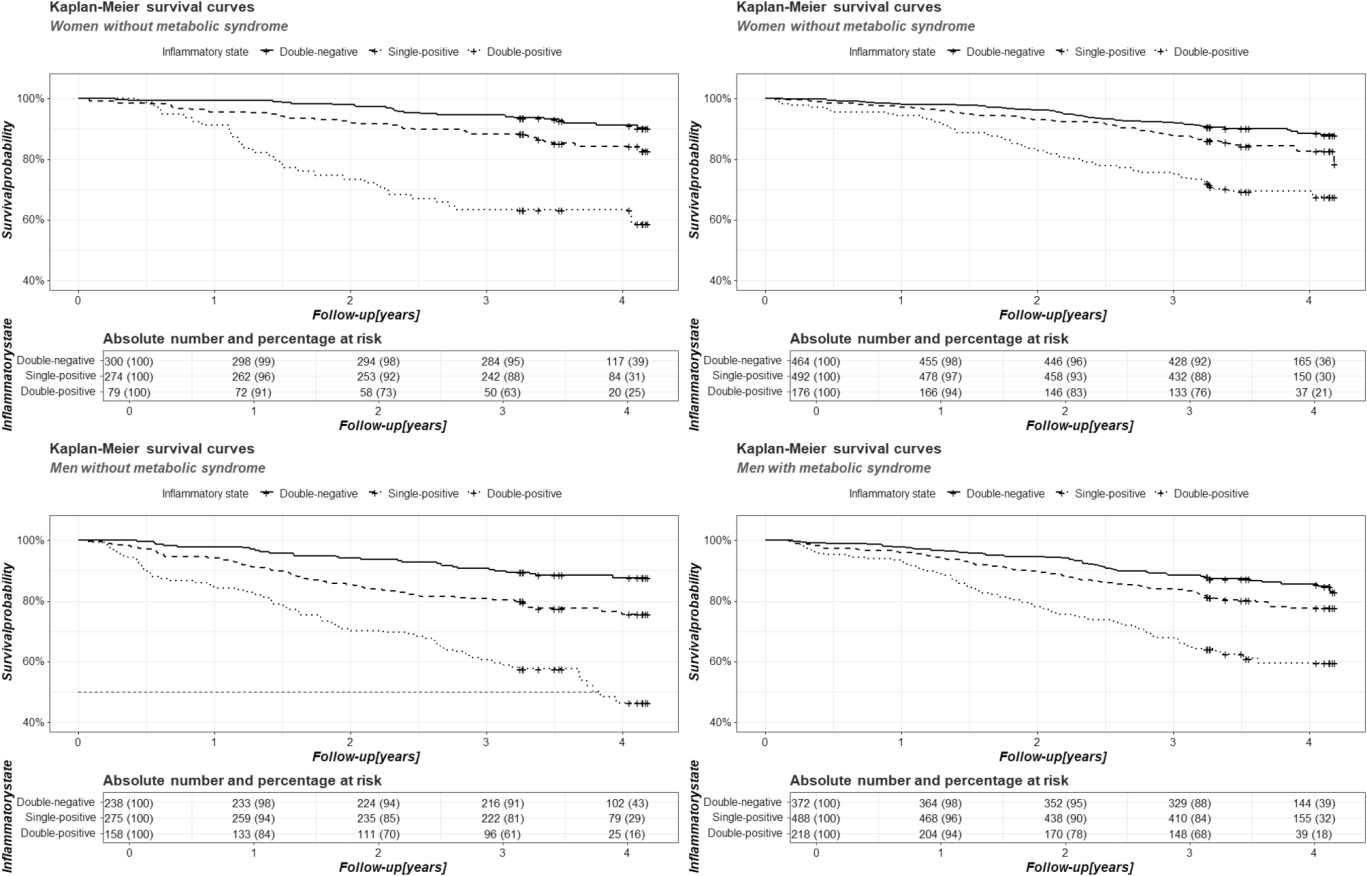
Kaplan-Meier curves based on the presence of MS and inflammation categories, separately for women (left) and men (right)

Kaplan-Meier analysis showed no modifying effect of MS on survival for both men and women in subgroups stratified for inflammatory categories (Fig. 2). The inflammatory category effect on survival was already significant in the single-positive subgroups, and more marked for double-positive subgroups (with *p*-values at least < 0.05). The restricted mean survival times within specific inflammatory categories (double-negative, single-positive, and double-positive, respectively) in the subgroups without MS were 3.93, 3.61, and 3.02 years for men; and 4.04, 3.84, and 3.19 years for women. Similarly, the restricted mean survival times in subgroups with MS were 3.89, 3.72, and 3.28 years for men and 3.97. 3.86, and 3.48 years for women.

In the univariate Cox regression analysis, factors that reduced survival included: advanced age, IADL disability, poor nutritional status, positive inflammatory categories, diabetes mellitus, CAD, past stroke, heart failure, and impaired kidney function. On the other hand, the elements that have a protective influence on survival were: visceral obesity, albumin level ≥ 40 g/L, hypertriglyceridemia, hypercholesterolemia, and prediabetes (but only in men) – Table 2.

**Table 2:** Results of univariate Cox regression analyses for the subject’s survival

	Men		Women	
	HR	95% CI	HR	95% CI
Age ≥ 80 yrs	4.48^#^	(3.65–5.49)	7.60^#^	(5.80–9.95)
IADL < 24 pts	5.92^#^	(4.77–7.35)	6.34^#^	(4.86–8.27)
MNA < 12 pts	3.93^#^	(3.21–4.82)	4.77^#^	(3.68–6.18)
Negative inflammatory subgroup	Ref.		Ref.	
Single-positive inflammatory subgroup	1.75^#^	(1.34–2.29)	1.71^#^	(1.28–2.29)
Double-positive inflammatory subgroup	3.98^#^	(3.04–5.21)	4.17^#^	(3.05–5.71)
Metabolic syndrome	0.88	(0.72–1.07)	1.12	(0.87–1.43)
Visceral obesity	0.59^#^	(0.48–0.73)	0.49^#^	(0.36–0.66)
Normal glucose control	Ref.		Ref.	
Prediabetes	0.74*	(0.56–0.97)	1.10	(0.80–1.51)
Diabetes mellitus	1.29*	(1.04–1.60)	1.55^#^	(1.19–2.04)
Hypertriglyceridemia	0.65^#^	(0.51–0.84)	0.77	(0.59–1.02)
Hypercholesterolemia	0.60^#^	(0.49–0.73)	0.45^#^	(0.35–0.58)
Hypertension	0.99	(0.79–1.24)	1.21	(0.91–1.63)
Coronary artery disease	1.26*	(1.03–1.55)	1.84^#^	(1.44–2.36)
Past stroke	2.07^#^	(1.60–2.69)	2.06^#^	(1.46–2.92)
Heart failure	1.47^#^	(1.17–1.84)	3.05^#^	(2.35–3.95)
Cancer survivors	1.45*	(1.08–1.95)	1.05	(0.71–1.57)
Use of statin	0.88	(0.72–1.08)	1.03	(0.81–1.32)
Albumin ≥ 40 g/L	0.29^#^	(0.24–0.33)	0.26^#^	(0.20–0.33)
eGFR < 45 ml/min/1.73m^2^	3.98^#^	(3.01–5.25)	4.13^#^	(3.11–5.47)

Statistical significance: *p < 0.05; **p < 0.01; ^#^*p* < 0.001

MNA – Mini Nutritional Assessment scale, IADL – Instrumental Activities of Daily Living scale, eGFR – estimated glomerular filtration rate, HR – hazard ratio, yrs – years, pts – points, CI – confidence interval, Ref. **–** reference

Each significant variable from the univariate analysis was included in the multivariate analysis, which delivered the following independent risk factors: oldest-old age, disability, poor nutritional status, single-positive, and double-positive inflammatory categories, impaired kidney function, and heart failure (only in women). Contrarily, the protective factors for survival in this analysis were visceral obesity for both genders and albumin levels ≥ 40 g/L only in men (Table 3).

**Table 3:** Results of multivariate Cox regression analyses for the subject’s survival

	Men		Women	
	HR	95% CI	HR	95% CI
Age ≥ 80 years	2.15^#^	(1.70–2.72)	3.33^#^	(2.38–4.65)
IADL < 24 pts	2.95^#^	(2.30–3.79)	1.87^#^	(1.33–2.63)
MNA < 12 pts	2.03^#^	(1.63–2.52)	2.00^#^	(1.48–2.70)
Negative inflammatory subgroup	Ref.		Ref.	
Single positive inflammatory subgroup	1.34^*^	(1.02–1.77)	1.44^*^	(1.04–1.99)
Double positive inflammatory subgroup	2.36^#^	(1.77–3.14)	2.99^#^	(2.09–4.27)
Metabolic syndrome	NS		NS	
Visceral obesity	0.79^*^	(0.63–0.98)	0.49^#^	(0.35–0.69)
Normal glucose control	Ref.		Ref.	
Prediabetes	NS		NS	
Diabetes mellitus	NS		NS	
Hypertriglyceridemia	NS		NS	
Hypercholesterolemia	NS		NS	
Coronary artery disease	NS		NS	
Heart failure	NS		1.65^#^	(1.24–2.20)
Cancer survivors	NS		NS	
eGFR < 45 ml/min/1.73m^2^	1.83^#^	(1.36–2.47)	1.76^*^	(1.28–2.42)
Albumin ≥ 40 mg/dL	0.73^**^	(0.57–0.92)	NS	

Statistical significance: *p < 0.05; **p < 0.01; ^#^*p* < 0.001; NS – not significant

MNA – Mini Nutritional Assessment scale, IADL – Instrumental Activities of Daily Living scale, eGFR – estimated glomerular filtration rate, HR – hazard ratio, yrs – years, pts – points, CI - confidence interval, Ref. **–** reference,

The variable ‘Past stroke’ was not included in the models due to the low frequency of episodes in the study population

After adjustment for the confounders, both the moderate inflammatory state (single-positive group) and enhanced inflammatory state (double-negative group) predicted the increased all-cause mortality.

### Correlation between CRP and PTX3

In the entire cohort, a very weak correlation between plasma CRP and PTX-3 concentrations was observed (ρ = 0.113; *p* < 0.001). Of note, the correlation was stronger in participants with CRP > 3 mg/dL then in those with lower CRP values (ρ = 0.214; *p* < 0.001) The strongest relatively were found in individuals with CRP > 3 mg/dL and either with poor nutritional status (ρ = 0.275; *p* < 0.001) or without MS (ρ = 0.272; *p* < 0.001). Overall, the strength of these correlations was weak (ρ < 0.3).

There was no meaningful correlation between statin use and PTX3 levels (stratified through inflammatory markers subgroups Spearman rank correlation ρ = 0.044; *p* < 0.001). Similar PTX-3 levels were observed in subjects using and not using statins [2.06 (1.60; 2.59) vs. 1.94 (1.53; 2.53); *p* = 0.21].

## Discussion

As described above, MS and plasma PTX-3 levels are associated with increased CV and all-cause mortality among subjects aged 65 years and over [2, 20]. In addition, some studies suggested an association between plasma PTX-3 levels and MS components and their severity [21–24]. Nevertheless, to the best of our knowledge, this is the first study that assessed plasma PTX-3 levels as a marker of mortality depending on the occurrence of MS in older populations. We show that plasma PTX-3 levels may play a role as a predictor of all-cause mortality in the general population of persons aged 65 years and older independently of the occurrence of MS. We found that mortality for single-positive, and double-positive subgroups was increasing both for men and women, independently of MS occurrence, with the highest rates observed in double-positive subgroups without MS. Moreover, Kaplan-Meier analysis showed no effect of MS on survival both for men and women in subgroups within specific inflammatory categories. In addition, contrary to previous studies conducted in middle-aged people [21–23] in our study group, there was no association between MS occurrence and plasma PTX-3 levels. Of interest, the severity of systemic inflammation corresponding to elevated concentrations of both PTX-3 and CRP was associated with older age, poor nutritional status, disability, lower serum albumin levels, and poorer excretory kidney function. Among the components of MS, only diabetes was more common in the subgroup with the most severe inflammation. Regarding the linking factors, diabetes mellitus is the most common factor of kidney failure [34]. Thus, our results suggested that with age, the importance of the components of MS as factors related to inflammation and influencing mortality may decrease. In contrast, the significance of the consequences of these components, such as chronic kidney disease, may increase. This hypothesis is partially supported by the study showing that the increased plasma PTX-3 levels predicted all-cause mortality in dialysis patients in a survival model adjusted for CRP, IL-6, TNF-alfa, leptin, and adiponectin levels [35]. Moreover, a recently published study analyzed subphenotypes of frailty in lung transplant candidates and found that subfenotype 2, characterized by systemic and innate inflammation (higher PTX3 as well as IL-6, CRP, TNF-R1, and IL-1RA), mitochondrial stress (higher GDF-15 and FGF-21), sarcopenia, malnutrition, and lower hemoglobin and walk distance, had a higher level of disability and higher risk of waitlist delisting or death [36]. Of note, our previous study showed that higher plasma FGF-21 concentration is an independent predictor of all-cause mortality in the general population of older adults [37]. The present study found the same for plasma PTX-3 levels, expanding the list of predictors of overall mortality.

In our study, elevated plasma PTX-3 and CRP concentrations indicating systemic inflammation were associated with poor nutritional status and lower albumin levels. The risk factors of malnutrition in the older population are complex and include socioeconomic factors (level of education, unmarried status, living in a rural area, subjective loneliness, and self-reported poverty) [38, 39] as well as factors related to numerous chronic diseases, especially cancer, heart failure, and CKD [40]. However, it should be noted that of these three common causes of malnutrition, the percentage of people with renal dysfunction increased significantly with the severity of inflammation in both subgroups with and without MS regardless of sex. Whereas the percentage of people with heart failure increased only in the MS subgroup and the rate of cancer survivors was similar regardless of severity of inflammation in both subgroups. This observation strengthens our previous hypothesis that in old age, not MS but its long-term consequences that significantly impact survival. The association between systemic inflammation and malnutrition seems bidirectional. As described above, PTX-3 is a more specific marker of vascular inflammation than other members of the pentraxin family [5]. In turn, components of MS are important factors in the development of atherosclerosis and its consequences [4]. Increasing systemic inflammation, inadequate dietary intake, and undernutrition-driven catabolism are key drivers of disease-related malnutrition [40]. On the other hand, insufficient dietary intake related to ‘anorexia of aging’ itself is a cause of immune system dysfunction and gut mucosal damage [41]. Malnutrition, regardless of its cause, is associated with increased morbidity and mortality both in acute and chronic disease [42]. However, it should be noted that after adjusting for the confounders, both the moderate and enhanced inflammatory states predicted increased all-cause mortality. Thus, both hepatic and vascular inflammatory responses (related to increased CRP and PTX-3 levels [6, 7], respectively) are associated with the increased risk of death. Due to the lack of data on the direct causes of death, a broader explanation of these mechanisms is not possible. Contrary to previously published studies [43, 44], we did not observe an association between plasma PTX-3 levels and treatment with statins.

From a clinical point of view, an important implication of our study is the determination of sex-specific cut-off points for PTX-3 associated with an increased risk of death during the 4.19-year follow-up at 2.07 ng/mL for men and 2.23 ng/mL for women. These points are significantly higher than the cut-off point calculated for differentiation between mild and severe MS in the middle-aged population (0.107 ng/mL) [21]. Nevertheless, the risk of cardiac events in patients with chronic HF was increased in groups with PTX-3 > 3.64 ng/mL [15]. Moreover, the meta-analysis of 15 studies, including 11,365 participants with coronary artery disease, showed that the adjusted odds ratios for composite poor outcomes increased by 32% per 1 ng/mL PTX-3 increment [19]. However, despite the fact that these cut-off points have been determined and that reliable and relatively inexpensive tests are available, introducing PTX-3 testing into everyday clinical practice for stratification of mortality risk requires further prospective studies to confirm our results. Furthermore, it is necessary to select patient groups in which performing such tests would be of particular importance, if therapies aimed at modulating PTX-3 secretion or inhibiting its action are available. Therefore, at this point, our study is a contribution to existing knowledge and indicates directions for further research, but it cannot be treated as recommendation to implement PTX-3 testing into everyday clinical practice.

The main limitation of our study is the lack of data on the causes of death. Moreover, a cross-sectional study design precluded analysis of inflammation severity changes with time and health conditions.

Our study’s strength is its population-based design, allowing us to generalize its results to older Caucasian populations.

## Conclusion

Our study suggests that in the age-advanced Caucasian population, the inflammatory status with increased plasma levels of both PTX-3 and CRP is associated with a higher risk of all-cause mortality, regardless of the occurrence of MS. However, due to the retrospective study design, these results require confirmation in prospective studies with an analysis of the underlying causes of death.

## Electronic supplementary material

Below is the link to the electronic supplementary material.

Supplementary Material 1

## Data Availability

No datasets were generated or analysed during the current study.

## Abbreviations

BMI: Body mass index
CAD: Coronary artery disease
CHD: Coronary heart disease
CVD: Cardiovascular disease
eGFR: estimated glomerular filtration rate
GFR: Glomerular filtration rate
HDL: High-density lipoprotein
HF: Heart failure
HFpEF: Heart failure with preserved ejection fraction
HR: Hazard ratio
hs-CRP: high-sensitivity C-reactive protein
IADL: Instrumental Activities of Daily Living
LDL: Low-density lipoprotein
MNA: Mini Nutritional Assessment, MS: metabolic syndrome
NT-proBNF: N-terminal prohormone of brain natriuretic peptide
PTX-3: Pentraxin-3
