# Supplementary material for: Pentraxin-3 and C-reactive protein plasma levels predict survival in older adults with or without metabolic syndrome – results of the PolSenior2 substudy
Source: Immun Ageing. 2025 May 8;22:16. doi: 10.1186/s12979-025-00509-9 (PMC12060356; doi:10.1186/s12979-025-00509-9)
Supplement: Supplementary file 1 — Supplementary Material 1 [file 12979_2025_509_MOESM1_ESM.docx]

Supplementary Table 1: Baseline clinical features of the study population based on the presence of metabolic syndrome and inflammation categories (N=3534; 1749 – 49.5% women, 1785 – 50.5% men).

|  | Metabolic syndrome absent | | | | Metabolic syndrome present | | | |
| --- | --- | --- | --- | --- | --- | --- | --- | --- |
| Inflammatory state: | Negative | Single positive | Double positive | p | Negative | Single positive | Double positive | p |
| Men |  |  |  |  |  |  |  |  |
| N (%) | 238 (35.5%) | 275 (41.0%) | 158 (23.5%) |  | 372 (35.5%) | 488 (45.3%) | 218 (20.2%) |  |
| Age [yrs] | 71 (65; 80) | 75 (67; 85)** | 78 (70; 87)^#^ | <0.001 | 73 (67; 80) | 74 (68; 81) | 75 (69; 83)* | <0.05 |
| BMI [kg/m^2^] | 26.0 (23.8; 28.5) | 25.7 (23.5; 28.3) | 24.3 (22.4; 27.1)^#^ | <0.001 | 29.3 (26.8; 32.1) | 28.8 (26.2; 31.8) | 30.0 (26.7; 32.9) | <0.05 |
| Obesity, N(%) | 38 (16.2%) | 46 (17.2%) | 19 (12.7%) | 0.46 | 160 (43.1%) | 177 (37.0%) | 105 (50.0%) | <0.01 |
| Total cholesterol [mg/dL] | 195.0 (172.0; 220.0) | 183.0 (156.0; 213.0)* | 175.0 (147.0; 206.0)^#^ | <0.001 | 169.0 (144.0; 200.0) | 164.0 (136.0; 192.3) | 160.0 (133.3; 194.0) | <0.05 |
| HDL-cholesterol [mg/dL] | 53.2 (46.1; 62.2) | 51.4 (44.0; 61.6) | 49.8 (44.0; 58.8)* | <0.05 | 44.8 (38.8; 52.4) | 43.5 (37.4; 52.1) | 41.2 (36.0; 49.8)** | <0.01 |
| LDL-cholesterol [mg/dL] | 127.3 (102.6; 151.3) | 115.3 (91.6; 148.4) | 111.0 (85.7; 142.3)** | <0.01 | 103.0 (80.1; 129.3) | 96.8 (74.5; 131.5) | 98.6 (73.6; 132.5) | 0.44 |
| Triglycerides [mg/dL] | 99.0 (78.3; 126.0) | 99.0 (75.0; 119.0) | 96.0 (80.0; 116.0) | 0.35 | 129.0 (95.8; 175.0) | 128.5 (93.0; 182.0) | 119.0 (96.3; 160.5) | 0.27 |
| Women |  |  |  |  |  |  |  |  |
| N (%) | 300 (45.9%) | 274 (42.0%) | 79 (12.1%) |  | 464 (41.0%) | 492 (43.5%) | 176 (15.5%) |  |
| Age [yrs] | 69 (65; 78) | 71 (66; 80) | 77 (69; 87)^#^ | <0.001 | 74 (67; 81) | 74 (68; 82) | 77 (68; 85)* | <0.05 |
| BMI [kg/m^2^] | 25.8 (23.5; 28.5) | 26.7 (23.3; 30.0) | 25.1 (21.8; 30.3) | 0.061 | 29.3 (26.2; 32.3) | 30.3 (27.0; 34.0)^#^ | 31.1 (27.6; 34.4)^#^ | <0.001 |
| Obesity, N(%) | 47 (16.0%) | 68 (25.1%)* | 21 (27.3%)** | <0.05 | 202 (44.4%) | 255 (52.9%)** | 99 (58.6%)** | <0.01 |
| Total cholesterol [mg/dL] | 215.5 (188.0; 240.3) | 212.0 (186.0; 233.0) | 191.0 (161.0; 214.0)^#^ | <0.001 | 184.0 (155.0; 220.3) | 183.5 (151.0; 216.0) | 173.0 (147.5; 208.3) | 0.16 |
| HDL-cholesterol [mg/dL] | 64.9 (56.7; 73.5) | 62.8 (55.1; 72.9) | 57.2 (49.5; 66.9)^#^ | <0.001 | 52.3 (45.5; 60.9) | 49.8 (44.4; 58.9) | 46.2 (41.2; 55.0)^#^ | <0.001 |
| LDL-cholesterol [mg/dL] | 133.6 (106.3; 161.3) | 132.2 (107.7; 154.3) | 117.0 (91.8; 147.8)** | <0.01 | 107.8 (84.7; 145.8) | 114.5 (79.5; 148.8) | 107.3 (79.3; 142.2) | 0.66 |
| Triglycerides [mg/dL] | 107.0 (87.0; 127.3) | 104.0 (82.0; 127.0) | 106.0 (87.0; 127.0) | 0.87 | 136.5 (106.0; 175.0) | 130.0 (104.0; 173.0) | 137.0 (101.0; 188.0) | 0.48 |

Statistical significance vs group with negative inflammatory state: *p<0,05; **p<0.01; ^#^p<0.001; mean ± standard deviation, median (lower quartile; upper quartile)

N – number, BMI – Body Mass Index, HDL cholesterol – high-density lipoprotein cholesterol, LDL cholesterol – low-density lipoprotein cholesterol, yrs – years.

Supplementary Table 2: Baseline characteristics of men (N = 1785) stratified by the presence or absence of metabolic syndrome (MS) and inflammation categories.

|  | MS absent | | | | MS present | | | |
| --- | --- | --- | --- | --- | --- | --- | --- | --- |
| Inflammatory categories | Negative | Single-positive | Double-positive | p | Negative | Single-positive | Double-positive | p |
| N (%) | 238 (35.5%) | 275 (41.0%) | 158 (23.5%) |  | 372 (35.5%) | 488 (45.3%) | 218 (20.2%) |  |
| PTX3 [ng/mL] | 1.60 (1.35; 1.79) | 2.31 (1.94; 2.84) | 2.82 (2.30; 3.45) | – | 1.60 (1.34; 1.83) | 2.23 (1.74; 2.66) | 2.70 (2.35; 3.25) | – |
| CRP [mg/dL] | 1.08 (0.68; 1.85) | 1.86 (0.87; 3.56) | 6.87 (4.28; 12.30) | – | 1.06 (0.67; 1.82) | 2.02 (0.94; 4.43) | 6.18 (4.11; 12.2) | – |
| Age ≥ 80 yrs, N(%) | 66 (27.7%) | 104 (37.8%)* | 70 (44.3%)^#^ | <0.01 | 97 (26.1%) | 151 (30.9%) | 82 (37.6%)^#^ | <0.05 |
| Visceral obesity, N(%) | 131 (55.0%) | 148 (53.8%) | 73 (46.2%) | 0.19 | 349 (93.8%) | 443 (90.8%) | 197 (90.4%) | 0.20 |
| MNA < 12 pts, N(%) | 44 (19.4%) | 75 (29.3%) | 70 (46.1%)^#^ | <0.001 | 72 (20.2%) | 108 (22.9%) | 73 (34.6%)^#^ | <0.001 |
| IADL < 24 pts, N(%) | 69 (29.2%) | 100 (36.6%) | 80 (50.6%)^#^ | <0.001 | 87 (23.4%) | 175 (35.9%)# | 90 (41.3%)^#^ | <0.001 |
| Hypertension, N(%) | 112 (47.3%) | 147 (53.5%) | 97 (61.4%)** | <0.05 | 332 (89.2%) | 446 (91.4%) | 202 (93.1%) | 0.27 |
| Coronary artery disease, N(%) | 52 (22.2%) | 68 (25.3%) | 45 (29.0%) | 0.31 | 132 (36.3%) | 204 (42.5%) | 99 (45.6%) | 0.057 |
| Stroke, N(%) | 13 (5.5%) | 20 (7.3%) | 21 (13.5%)** | <0.05 | 37 (9.9%) | 62 (12.8%) | 25 (11.5%) | 0.43 |
| Heart failure, N(%) | 33 (14.3%) | 35 (13.9%) | 24 (16.6%) | 0.76 | 79 (22.4%) | 136 (29.2%)* | 67 (32.1%)* | <0.05 |
| Cancer survivors, N(%) | 20 (8.4%) | 22 (8.0%) | 19 (12.0%) | 0.34 | 31 (8.3%) | 52 (10.7%) | 17 (7.8%) | 0.34 |
| Normal glucose control, N(%) | 190 (79.8%) | 225 (81.8%) | 128 (81.0%) | 0.78 | 110 (29.6%) | 143 (29.3%) | 41 (18.8%)^#^ | <0.001 |
| Prediabetes, N(%) | 33 (13.9%) | 30 (10.9%) | 17 (10.8%) |  | 137 (36.8%) | 134 (27.5%) | 62 (28.4%) |  |
| Diabetes mellitus, N(%) | 15 (6.3%) | 20 (7.3%) | 13 (8.2%) |  | 125 (33.6%) | 211 (43.2%)* | 115 (52.8%)^#^ |  |
| eGFR < 45 ml/min/1.73m^2^, N(%) | 3 (1.3%) | 9 (3.3%) | 14 (8.9%)^#^ | <0.001 | 15 (4.0%) | 30 (6.1%) | 27 (12.4%)^#^ | <0.001 |
| Albumin < 40 g/L, N(%) | 22 (9.2%) | 46 (16.7%)* | 45 (28.5%)^#^ | <0.001 | 25 (6.7%) | 60 (12.3%)** | 48 (22.0%)^#^ | <0.001 |
| Fasting glucose [mg/dL] | 93.0 (87.0; 97.0) | 92.0 (87.0; 98.0) | 91.0 (86.3; 96.0) | 0.38 | 104.0 (95.0; 117.0) | 103.0 (94.0; 122.0) | 107.0 (97.0; 124.0) | 0.075 |
| HDL-cholesterol [mg/dL] | 53.2 (46.1; 62.2) | 51.4 (44.0; 61.6) | 49.8 (44.0; 58.8)* | <0.05 | 44.8 (38.8; 52.4) | 43.5 (37.4; 52.1) | 41.2 (36.0; 49.8)** | <0.01 |
| Triglycerides [mg/dL] | 99.0 (78.3; 126.0) | 99.0 (75.0; 119.0) | 96.0 (80.0; 116.0) | 0.35 | 129.0 (95.8; 175.0) | 128.5 (93.0; 182.0) | 119.0 (96.3; 160.5) | 0.27 |
| Hypercholesterolemia, N(%) | 161 (67.6%) | 158 (57.5%)* | 77 (48.7%)# | <0.001 | 286 (76.9%) | 385 (78.9%) | 158 (72.5%) | 0.17 |
| Hypertriglyceridemia, N(%) | 21 (8.8%) | 16 (5.8%) | 5 (3.2%) | 0.069 | 49 (40.1%) | 195 (40.0%) | 65 (29.8%)* | <0.05 |
| Statins, N(%) | 34 (14.3%) | 45 (16.4%) | 19 (12.0%) | 0.46 | 203 (54.6%) | 280 (57.4%) | 113 (51.8%) | 0.37 |
| Fibrates, N(%) | 0 (0.0%) | 2 (0.7%) | 0 (0.0%) | 0.52 | 13 (3.5%) | 13 (2.7%) | 11 (5.0%) | 0.27 |
| Death, N(%) | 28 (11.76%) | 63 (22.91%) | 73 (46.20%) | < 0.001 | 52 (13.98%) | 101 (20.70%) | 84 (38.53%) | < 0.001 |
| Death rate per 1000 (95% CI) | 32.8 (22.8 – 47.6) | 70.4 (55.0 – 90.2) | 167.6 (133.2 – 210.8) |  | 39.7 (30.2 – 52.1) | 61.6 (50.7 – 74.8) | 130.3 (105.2 – 161.4) |  |
| HR (95% CI) | Ref. | 2.14 (1.37 – 3.35)** | 5.11 (3.30 – 7.91)^#^ |  | Ref. | 1.56 (1.11 – 2.17)* | 3.34 (2.36 – 4.72)^#^ |  |
| HR (95% CI) age-adjusted | Ref. | 1.88 (1.20 – 2.93)** | 4.38 (2.82 – 6.79)^#^ |  | Ref. | 1.44 (1.03 – 2.02)* | 2.95 (2.08 – 4.18)^#^ |  |

Statistical significance vs group with negative inflammatory state: *p<0,05; **p<0.01; ^#^p<0.001

N – number, PTX3 – pentraxin 3, CRP – C-reactive protein, yrs – years, MNA – Mini Nutritional Assessment scale, IADL – Instrumental Activities of Daily Living scale, pts – points, , eGFR – estimated glomerular filtration rate, HDL cholesterol – high-density lipoprotein cholesterol, CI – confidence interval, HR – hazard ratio, Ref. **–** reference**.**

Supplementary Table 3: Baseline characteristics of women (N = 1749) stratified by the presence or absence of metabolic syndrome (MS) and inflammation categories.

|  | MS absent | | | | MS present | | | |
| --- | --- | --- | --- | --- | --- | --- | --- | --- |
| Inflammatory categories | Negative | Single-positive | Double-positive | p | Negative | Single-positive | Double-positive | p |
| N (%) | 300 (45.9%) | 274 (42.0%) | 79 (12.1%) |  | 464 (41.0%) | 492 (43.5%) | 176 (15.5%) |  |
| PTX3 [ng/mL] | 1.67 (1.35; 1.91) | 2.24 (1.68; 2.65) | 2.90 (2.45; 3.55) | – | 1.62 (1.36; 1.93) | 2.05 (1.59; 2.68) | 2.90 (2.54; 3.44) | – |
| CRP [mg/dL] | 1.37 (0.71; 2.06) | 2.92 (1.29; 4.72) | 6.73 (4.41; 11.5) | – | 1.40 (0.83; 2.09) | 3.42 (1.62; 5.92) | 7.02 (4.48; 10.50) | – |
| Age ≥ 80 years, N(%) | 69 (23.0%) | 70 (25.5%) | 35 (44.3%)^#^ | <0.001 | 132 (28.4%) | 176 (35.8%)* | 76 (43.2%)^#^ | <0.01 |
| Visceral obesity, N(%) | 218 (72.7%) | 209 (76.3%) | 54 (68.4%) | 0.32 | 446 (96.1%) | 478 (97.2%) | 171 (97.2%) | 0.63 |
| MNA < 12 pts, N(%) | 65 (22.8%) | 90 (34.6%)** | 38 (52.1%)^#^ | <0.001 | 106 (23.8%) | 156 (32.8%)** | 67 (39.4%)^#^ | <0.001 |
| IADL < 24 pts, N(%) | 74 (24.7%) | 77 (28.1%) | 36 (45.6%)^#^ | 0.001 | 131 (28.3%) | 187 (38.0%)# | 86 (49.1%)^#^ | <0.001 |
| Hypertension, N(%) | 133 (44.5%) | 151 (55.3%)* | 41 (51.9%) | <0.05 | 426 (91.8%) | 457 (92.9%) | 165 (93.8%) | 0.66 |
| Coronary artery disease, N(%) | 48 (16.2%) | 48 (17.7%) | 13 (17.3%) | 0.89 | 127 (27.9%) | 136 (28.0%) | 47 (27.8%) | >0.99 |
| Stroke, N(%) | 5 (5.0%) | 14 (5.1%) | 5 (6.4%) | 0.80 | 35 (7.6%) | 40 (8.2%) | 24 (13.9%)* | <0.05 |
| Heart failure, N(%) | 23 (7.9%) | 30 (11.3%) | 11 (14.5%) | 0.16 | 77 (17.3%) | 85 (18.4%) | 43 (27.0%)** | <0.05 |
| Cancer survivors, N(%) | 24 (8.0%) | 32 (11.7%) | 7 (8.9%) | 0.32 | 47 (10.1%) | 34 (6.9%) | 23 (13.1%) | < 0.05 |
| Normal glucose control, N(%) | 255 (85.0%) | 248 (90.5%) | 71 (89.9%) | 0.35 | 207 (44.6%) | 189 (38.4%) | 58 (33.0%)* | <0.01 |
| Prediabetes, N(%) | 28 (9.3%) | 16 (5.8%) | 5 (6.3%) |  | 123 (26.5%) | 136 (27.6%) | 42 (23.9%) |  |
| Diabetes mellitus, N(%) | 17 (5.7%) | 10 (3.6%) | 3 (3.8%) |  | 134 (28.9%) | 167 (33.9%) | 76 (43.2%)** |  |
| eGFR < 45 ml/min/1.73m^2^, N(%) | 9 (3.0%) | 14 (5.1%) | 10 (12.7%)^#^ | <0.01 | 32 (6.9%) | 49 (10.0%) | 32 (18.2%)^#^ | <0.001 |
| Albumin < 40 g/L, N(%) | 23 (7.7%) | 36 (13.1%)* | 26 (32.9%)^#^ | <0.001 | 47 (10.1%) | 59 (12.0%) | 38 (21.6%)^#^ | <0.001 |
| Fasting glucose [mg/dL] | 90.0 (85.0; 96.0) | 90.0 (85.0; 95.0) | 89.0 (83.0; 91.5)* | <0.05 | 99.0 (90.0; 109.3) | 101.0 (91.8; 113.0) | 106.0 (91.0; 122.0)** | <0.01 |
| HDL-cholesterol [mg/dL] | 64.9 (56.7; 73.5) | 62.8 (55.1; 72.9) | 57.2 (49.5; 66.9)^#^ | <0.001 | 52.3 (45.5; 60.9) | 49.8 (44.4; 58.9) | 46.2 (41.2; 55.0)^#^ | <0.001 |
| Triglycerides [mg/dL] | 107.0 (87.0; 127.3) | 104.0 (82.0; 127.0) | 106.0 (87.0; 127.0)** | 0.87 | 136.5 (106.0; 175.0) | 130.0 (104.0; 173.0) | 137.0 (101.0; 188.0) | 0.48 |
| Hypercholesterolemia, N(%) | 246 (82.0%) | 211 (77.0%) | 47 (59.5%)# | <0.001 | 387 (83.4%) | 405 (82.3%) | 132 (75.0%)* | <0.05 |
| Hypertriglyceridemia, N(%) | 21 (7.0%) | 21 (7.7%) | 2 (2.5%) | 0.27 | 193 (41.6%) | 199 (40.4%) | 84 (47.7%) | 0.24 |
| Statins, N(%) | 48 (16.0%) | 27 (9.9%) | 8 (10.1%) | 0.067 | 237 (51.1%) | 237 (48.2%) | 79 (44.9%) | 0.35 |
| Fibrates, N(%) | 1 (0.3%) | 0 (0.0%) | 0 (0.0%) | >0.99 | 13 (2.8%) | 14 (2.8%) | 7 (4.0%) | 0.71 |
| Death, N(%) | 24 (8.00%) | 41 (14.96%) | 30 (37.97%) | < 0.001 | 49 (10.56%) | 79 (16.06%) | 54 (30.68%) | < 0.001 |
| Death rate per 1000 (95% CI) | 22.0 (14.7 – 32.8) | 43.5 (32.0 – 59.0) | 130.7 (91.4 – 187.0) |  | 29.7 (22.5 – 39.3) | 46.5 (37.3 – 58.0) | 98.7 (75.6 – 128.8) |  |
| HR (95% CI) | Ref. | 1.98 (1.20 – 3.28)** | 6.11 (3.57 – 10.47)^#^ |  | Ref. | 1.57 (1.10 – 2.25)* | 3.40 (2.31 – 5.01)^#^ |  |
| HR (95% CI) age adj. | Ref. | 2.03 (1.23 – 3.37)** | 4.46 (2.59 – 7.68)^#^ |  | Ref. | 1.37 (0.96 – 1.96) | 2.82 (1.92 – 4.17)^#^ |  |

Statistical significance vs group with negative inflammatory state: *p<0,05; **p<0.01; ^#^p<0.001

N – number, PTX3 – pentraxin 3, CRP – C-reactive protein, yrs – years, MNA – Mini Nutritional Assessment scale, IADL – Instrumental Activities of Daily Living scale, pts – points, eGFR – estimated glomerular filtration rate, HDL cholesterol – high-density lipoprotein cholesterol, CI - confidence interval, HR – hazard ratio, Ref.  **–** reference**.**
